# Eligibility for Nirmatrelvir/Ritonavir Among Adults With an Acute Care COVID‐19 Encounter: A Retrospective Cohort Study From Alberta, Canada

**DOI:** 10.1002/pds.70302

**Published:** 2025-12-25

**Authors:** Sylvia Aponte‐Hao, Khanh Vu, Jason R. Randall, Karen J. B. Martins, Phuong Uyen Nguyen, Lynora Saxinger, Jenine Leal, Elissa Rennert‐May, Ellen Rafferty, Tyler Williamson, Scott W. Klarenbach

**Affiliations:** ^1^ Data and Research Services Alberta SPOR SUPPORT Unit Data Platform Calgary Alberta Canada; ^2^ Real World Evidence Unit, Faculty of Medicine and Dentistry University of Alberta Edmonton Alberta Canada; ^3^ Centre for Health Informatics, Cumming School of Medicine University of Calgary Calgary Alberta Canada; ^4^ Department of Medicine University of Alberta Edmonton Alberta Canada; ^5^ Department of Microbiology and Immunology University of Alberta Edmonton Alberta Canada; ^6^ Infection Prevention and Control Alberta Health Services Edmonton Alberta Canada; ^7^ Department of Community Health Sciences, Cumming School of Medicine University of Calgary Calgary Alberta Canada; ^8^ Department of Microbiology, Immunology, and Infectious Diseases, Cumming School of Medicine University of Calgary Calgary Alberta Canada; ^9^ O'Brien Institute for Public Health University of Calgary Calgary Alberta Canada; ^10^ Department of Medicine University of Calgary Calgary Alberta Canada; ^11^ Institute of Health Economics Edmonton Alberta Canada; ^12^ Libin Cardiovascular Institute, Department of Cardiac Science University of Calgary Calgary Alberta Canada; ^13^ Alberta Children's Hospital Research Institute University of Calgary Calgary Alberta Canada

**Keywords:** adult, COVID‐19, hospital, nirmatrelvir and ritonavir drug combination, public health, retrospective studies

## Abstract

**Purpose:**

To describe the characteristics of adults who had a severe coronavirus disease 2019 (COVID‐19) outcome during a timeframe that nirmatrelvir/ritonavir (NMV‐r; Paxlovid) was available, to assess medical eligibility and receipt of NMV‐r before presentation to acute care.

**Methods:**

Population‐level administrative data was used to describe adults (≥ 18 years) who had an acute care COVID‐19 encounter (hospitalization or emergency department visit with COVID‐19) between January 2022 and March 2023 in Alberta, Canada.

**Results:**

The 30 793 adults had an acute care COVID‐19 encounter; among the 8345 (27.1%) who were assessed as medically eligible for NMV‐r, 8111 (97.2%) did not receive outpatient NMV‐*r* ≤ 30 days before the encounter. During the acute care COVID‐19 encounter, the proportion who had an all‐cause death was 4.7% (received prior NMV‐r treatment) and 6.6% (did not) among those who were medically eligible for NMV‐r. Of those medically eligible for NMV‐r who were hospitalized (*n* = 4495), the mean length of stay was 9.7 days (SD 11.1) in NMV‐r treated and 15.3 days (SD 25.9) in untreated, and the proportion admitted to the intensive care unit was 2.1% in NMV‐r treated and 7.6% in untreated.

**Conclusion:**

Results from this real‐world study show that most adults with an acute care COVID‐19 encounter who were assessed as medically eligible for NMV‐r at the time of presentation had not received this outpatient treatment beforehand in Alberta, Canada, suggesting a potential care gap. If gaps are closed, strain on the acute healthcare system can be alleviated, with attendant improvement in other important patient outcomes.

## Key Points

- This study found that among adults who had an acute care encounter with coronavirus disease 2019 (COVID‐19) and were medically eligible for nirmatrelvir/ritonavir, most had not received this treatment within 30 days prior to presentation to acute care (97.2%) in Alberta, Canada.
- Results suggest that nirmatrelvir/ritonavir is largely underused in the target population, highlighting the need to identify and address barriers to early COVID‐19 oral antiviral treatment use for eligible high‐risk individuals to improve health outcomes and attendant health care resource use.
- If these care gaps are closed, individual health outcomes and strain on the acute healthcare system may be alleviated.

## Plain Language Summary

Nirmatrelvir/ritonavir (NMV‐r; Paxlovid) is an oral antiviral medication used for treating COVID‐19 in the outpatient setting. This drug has been shown to significantly reduce the risk of hospitalization and death in people at increased risk of severe COVID‐19 disease. We conducted a population‐level retrospective, observational, cohort study on almost 31 000 adults from Alberta, Canada, to understand the characteristics of people who had an acute care COVID‐19 encounter (went to the hospital or emergency department for COVID‐19) during a timeframe that NMV‐r was available for use. We found that among those who had an acute care COVID‐19 encounter, 27% were eligible to have received NMV‐r. Among those who were eligible, 97% had not received NMV‐r within 30 days before. Results of this study show that NMV‐r is largely underused in the target population, suggesting a potential care gap. If gaps are closed, strain on the acute healthcare system can be alleviated, with attendant improvement in other important patient outcomes.

## 1 Introduction

The coronavirus disease 2019 (COVID‐19) pandemic, caused by severe acute respiratory syndrome coronavirus 2 (SARS‐CoV‐2), has been one of the greatest threats to public health in the 21st century, with almost 779 million identified cases and over 7.1 million deaths reported worldwide (as of November 3, 2025) [1]. As a result, unprecedented strain has been placed on health care systems, including hospitals and intensive care units.

Antiviral therapies for the treatment of COVID‐19 and prevention of severe outcomes are valuable tools in the global pandemic response and long‐term endemicity. Several antiviral therapies have been approved by Health Canada for use among adults in the outpatient setting. Nirmatrelvir/ritonavir (NMV‐r; Paxlovid), the only orally administered antiviral therapy, became available in Canada on January 18, 2022 [2]. Remdesivir (administered intravenously) was approved for outpatient use on April 22, 2022 [3], but is rarely used in this setting in Canada [4]. Other therapies, including bamlanivimab, casirivimab/imdevimab, and sotrovimab (administered intravenously), as well as cilgavimab/tixagevimab (administered intramuscularly), had their authorizations for use in Canada canceled in 2024.

NMV‐r has been shown to significantly reduce the risk of hospitalization and mortality in individuals who are at high risk for progression to severe COVID‐19‐related outcomes [5, 6, 7]. In Canada, NMV‐r can be taken within 5 days of mild‐to‐moderate symptom onset in adults who test positive for SARS‐CoV‐2 and who are at high risk for developing severe COVID‐19‐related outcomes such as hospital admission, intensive care unit admission, and death [8]. Previous reports from the United States have shown that NMV‐r is largely underused in the target population [9, 10, 11]. Given this, an understanding of the characteristics of those who present to acute care with COVID‐19 in the era of available antiviral therapies may help inform the need of identifying potential care gaps in Canada. Such insights could guide strategies to improve treatment access and use, thereby reducing serious COVID‐19‐related outcomes and attendant healthcare system burden. The objective of this real‐world retrospective, observational cohort study was to describe the characteristics of adults who had an acute care COVID‐19 encounter (hospitalization or emergency department visit) during a timeframe that NMV‐r was available, to assess medical eligibility and receipt of NMV‐r before presentation to acute care.

## 2 Materials and Methods

Ethics approval was received from the University of Alberta Research Ethics Board (Pro00132799) that granted an exemption from requiring written informed consent (a waiver of consent was applied). Data custodian approvals were received from Alberta Health and Alberta Health Services (AHS; the largest single health authority in the province during this study) for the use of administrative health data. This study was reported according to the REporting of studies Conducted using Observational Routinely‐collected health Data (RECORD) guidelines [12].

### 2.1 Study Design

This real‐world retrospective, observational, population‐based cohort study was conducted using administrative health data from Alberta between January 18, 2012, and June 30, 2023. It encompassed a 14.5 month inclusion period from January 18, 2022 (first available date for use of NMV‐r in Canada) to March 31, 2023, up to a 10 year look‐back period for the assessment of baseline characteristics, and a 3 month extension after the inclusion period to encompass the duration of the acute care encounter. See Figure S1 for a graphical depiction of the study design.

### 2.2 Data Source

Canadian provinces have single‐payer health systems and provide publicly funded medically necessary care for all residents. While the majority of prescription drugs are not publicly funded, the Public Health Agency of Canada procured, allocated, and paid for NMV‐r during the inclusion period of this study. In Alberta, the fourth most populous province in Canada (4.5–4.7 million people between 2022 and 2023), healthcare is administered under the Alberta Health Care Insurance Plan (AHCIP), of which over 99% of Albertans participate [13, 14].

A person‐level linked (using a unique individual identifier [Personal Health Number]) data extract was created from the following listed databases by the data custodians and provided to the researchers (who conducted the analysis within the secure data custodian environment). The Population Registry contains demographic information for all Albertans with AHCIP coverage; elements include migration in and out of the province, and birth and death indicators. The Discharge Abstract Database (DAD) and National Ambulatory Care Reporting System (NACRS) include information on all individuals within the province who are discharged from hospitals and facility‐based ambulatory care clinics including emergency departments (ED), respectively; a most responsible diagnostic field and secondary fields are available and use International Classification of Disease‐Version 10‐Canadian Enhancement (ICD‐10‐CA) codes. The Practitioner Claims database includes information on physician billing in the province; up to three ICD‐Version 9 Clinical Modification (ICD‐9‐CM) diagnostic codes can be listed per visit. The Laboratory Information System includes laboratory test results collected by AHS. The Vital Statistics database contains information on births and deaths in the province reported to Alberta Health. The Pharmaceutical Information Network (PIN) contains information on all community‐dispensed prescription medications from pharmacies (regardless of payer); approximately 95% of all pharmacists in the province submit records to this database. The Alberta Cancer Registry collects information on demographics, tumor characteristics, and primary treatment from all individuals who received treatment in the province at the time of their initial cancer diagnosis. The Immunization and Adverse Reactions to Immunizations database contains records for vaccinations within the province, including data from AHS and pharmacist immunizers. The Alberta Continuing Care Information System contains information on residents living in a long‐term care facility and designated supportive living. Data is submitted to the Canadian Institute for Health Information who ensure the quality of the information within their data holdings. Additionally, data from DAD and NACRS are recorded by health information management coding professionals who perform regular data quality reviews and assurances; the face validity of diagnostic codes within the Practitioner Claims database has been found to be substantially high [15]; PIN is a primary dataset for community drug utilization reviews for quality improvement, research, and evaluations within Alberta Health Services. Records that were duplicates or contained an invalid Personal Health Number were discarded by the data custodians. Variables were checked for missing data and inconsistencies by the researchers, and inconsistent data were corrected using data logic or information majority.

### 2.3 Cohort Selection

Eligibility criteria for the cohort included those who: (1) had ≥ 1 hospitalization or ED visit with COVID‐19 (ICD‐10‐CA U07.1 or U07.3 located within any diagnostic field [16]; those who had post‐admission acquired COVID‐19 were excluded) during the inclusion period (January 18, 2022 to March 31, 2023), (2) were aged ≥ 18 years on the date of the first hospitalization or ED visit with COVID‐19 during the inclusion period (index date), and (3) had AHCIP coverage for ≥ 2 years before the index date and ≥ 3 months after the index date or until death, whichever occurred first. Individuals who had an index ED visit with COVID‐19 and were subsequently admitted to the hospital with COVID‐19 ≤ 7 days after were assigned an index date corresponding to the date of hospital admission. The codes used to identify individuals who had an acute care encounter with COVID‐19 have been previously validated in Alberta and resulted in high sensitivity (82.5%) and positive predictive value (92.9%) [16].

Those in the cohort who met medical eligibility criteria for NMV‐r (Table S2) were grouped based on whether they received a dispensation for NMV‐*r* ≤ 30 days before the index date or did not [17]. In the absence of symptom onset data in this study, a 30 day look back period was used to maximize the capture of outpatient NMV‐r dispensations plausibly related to the acute care COVID‐19 encounter; this was based on the typical median time and maximal clinical window reported and used in the literature between symptom onset and hospital admission [17, 18, 19, 20, 21].

### 2.4 Study Measures

Demographic characteristics recorded on the index date included age, sex, urban/rural residence (determined by the second digit of the postal code), and those residing within long‐term care or supportive living. Socioeconomic status (material and social deprivation indices) was measured using the Pampalon deprivation index that includes a material deprivation index (MDI; based on education, employment status, and average income) and a social deprivation index (SDI: based on living status, marital status, and number of parents in the household) [22]; components are derived from Canadian census data at the dissemination area level that is linkable to postal code, and presented based on quintiles from most well‐off (quintile 1) to most deprived (quintile 5) that represent the Alberta general population.

Clinical characteristics included the Charlson Comorbidity Index score that was measured during the 2 year pre‐index period (Table S1) [23, 24], and health conditions that were eligible for NMV‐r in Alberta during the inclusion period (Table S2 details the eligibility criteria [25, 26, 27, 28, 29, 30, 31]). A lookback period of up to 10 years before the index date, depending on the health condition and individual AHCIP coverage (required to have ≥ 2 years before the index date), was applied to identify individuals living with asthma, chronic kidney disease, chronic obstructive pulmonary disorder [COPD], congestive heart failure, diabetes and taking medication for this condition, an immunocompromised state, obesity, and pregnancy (Table S3 details the case definitions used [4, 23, 27, 29, 32, 33, 34, 35, 36, 37, 38, 39, 40, 41, 42, 43, 44, 45, 46, 47]). The number of previous COVID‐19 vaccine doses received, and those who had a prior infection of SARS‐CoV‐2 (had ≥ 1 laboratory confirmed COVID‐19 infection, healthcare encounter with COVID‐19 [ICD‐10‐CA U07.1, U07.3], or community pharmacy dispensation for a COVID‐19 treatment [NMV‐r, bamlanivimab, sotrovimab, or casirivimab‐imdevimab]) were reported.

All‐cause death during the index acute care COVID‐19 encounter was reported. For those who had an index hospitalization, length of stay, and intensive care unit admission and associated length of stay was reported.

### 2.5 Statistical Analysis

Descriptive statistics included counts and percentages for categorical variables, and means and standard deviations (SD) for continuous variables. In accordance with data custodian privacy standards, variables with 1–9 individuals were reported as < 10 (associated proportions were presented based on the number 5); where applicable, other outcomes were censored (e.g., presented as a range; associated proportions were presented based on the mid number of the range) so the number of individuals within the small cell size variable could not be calculated and potentially identified [48]. Analysis was performed using R (version 4.4.0) [49].

## 3 Results

### 3.1 Cohort Selection

**FIGURE 1:**
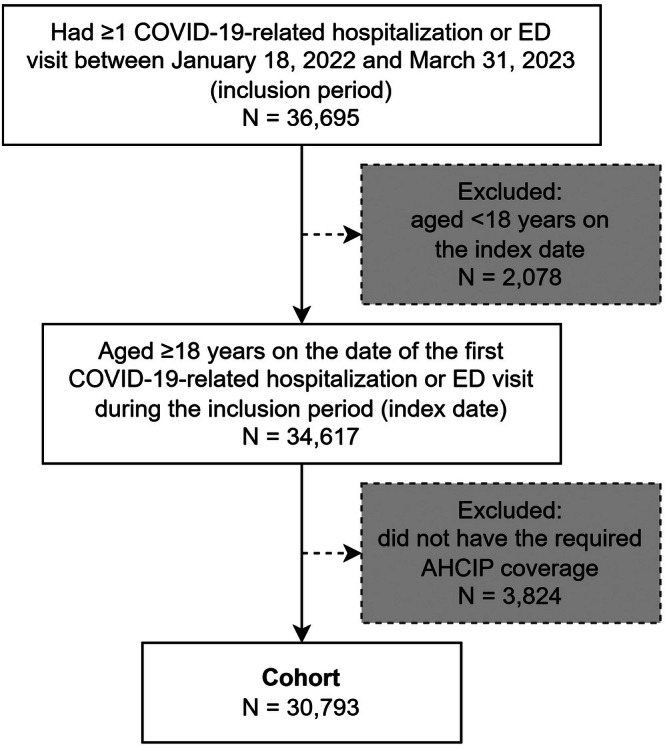
Cohort selection flow diagram. Abbreviations: Alberta Health Care Insurance Plan; COVID‐19, coronavirus disease 2019; ED, emergency department.

Among 36 695 individuals who had an acute care encounter with COVID‐19 during the inclusion period, a total of 30 793 adults met eligibility criteria and were included in the cohort (Figures 1 and S2 includes data linkage details); 13 112 (42.6%) individuals were hospitalized, and 17 681 (57.4%) visited the ED only.

### 3.2 Characteristics

A total of 8345 (27.1%; hospitalized: *n* = 4569, ED only: *n* = 3776) individuals within the cohort met medical eligibility criteria for NMV‐r, among whom 234 (2.8%) received this treatment ≤ 30 days before presentation to acute care and 8111 did not (97.2%) (Table 1). Among those who were medically eligible for NMV‐r, 5907 (70.8%) met eligibility by living with an immunocompromised condition (3.2% received NMV‐r treatment before the acute care encounter), 3660 (43.9%) by age/health condition/vaccination criteria (1.7% received NMV‐r treatment before the acute care encounter), and 293 (3.5%) by residing in long‐term care or supportive living (7.5% received NMV‐r treatment before the acute care encounter) (Table 1). Table S4 shows medical eligibility for NMV‐r according to the COVID‐19 diagnostic code location (primary [most responsible diagnostic field] or secondary) of the acute care encounter.

**TABLE 1:** Medical eligibility of the cohort for nirmatrelvir/ritonavir.

	Total	Index acute care encounter type	
		Hospitalized	ED only
	*n* = 30 793	*n* = 13 112	*n* = 17 681
Medically eligible for nirmatrelvir/ritonavir, *n* (%)	8345 (27.1%)	4569 (34.8%)	3776 (21.4%)
Overall			
Received nirmatrelvir/ritonavir	234 (2.8%)	74 (1.6%)	160 (4.2%)
Did not receive nirmatrelvir/ritonavir	8111 (97.2%)	4495 (98.4%)	3616 (95.8%)
According to eligibility criteria			
Immunocompromised	5907 (70.8%)	2918 (63.9%)	2989 (79.2%)
Received nirmatrelvir/ritonavir	191 (3.2%)	55 (1.9%)	136 (4.6%)
Did not receive nirmatrelvir/ritonavir	5716 (96.8%)	2863 (98.1%)	2853 (95.4%)
Age/health condition/vaccination status	3660 (43.9%)	2457 (53.8%)	1203 (31.9%)
Received nirmatrelvir/ritonavir	62 (1.7%)	21 (0.9%)	41 (3.4%)
Did not receive nirmatrelvir/ritonavir	3598 (98.3%)	2436 (99.1%)	1162 (96.6%)
Long‐term care/supportive living	293 (3.5%)	224 (4.9%)	69 (1.8%)
Received nirmatrelvir/ritonavir	22 (7.5%)	13–21 (~7.6%)	< 10 (~1.4%)
Did not receive nirmatrelvir/ritonavir	271 (92.5%)	203–211 (~92.4%)	60–68 (~98.6%)

Abbreviation: ED, emergency department.

The mean age of the cohort was 60 (SD 21) years (Table 2); those who were hospitalized with COVID‐19 were more likely to be older than those who visited the ED only (hospitalized: 70 [SD 19] years; ED only: 52 [SD 20] years) (Table S5). The majority were female (55.4%), and most lived in an urban area (78.6%) (Table 2). Socioeconomic status was presented based on quintiles that represent the Alberta general population (five groups with 20% in each); comparatively, the cohort was less likely to be socioeconomically well‐off (MDI 1 quintile: 15.1%; SDI 1 quintile: 13.6%) and more likely to be deprived (MDI 5 quintile: 25.1%; SDI 5 quintile: 29.0%) (Table 2).

**TABLE 2:** Baseline demographic characteristics of the total cohort.

	Total	Medically eligible for NMV‐r	
		*n* = 8345	
		Received treatment	Did not receive treatment
	*n* = 30 793	*n* = 234	*n* = 8111
Age, years			
Mean (SD)	60 (21)	66 (18)	64 (20)
Category, *n* (%)			
18–49	10 812 (35.1%)	48 (20.5%)	1945 (24.0%)
50–59	3730 (12.1%)	23 (9.8%)	943 (11.6%)
60–69	4708 (15.3%)	51 (21.8%)	1552 (19.1%)
≥ 70	11 543 (37.5%)	112 (47.9%)	3671 (45.3%)
Sex, *n* (%)			
Female	17 057 (55.4%)	136 (58.1%)	4769 (58.8%)
Male	13 736 (44.6%)	98 (41.9%)	3342 (41.2%)
Residence, *n* (%)			
Urban	24 198 (78.6%)	172 (73.5%)	6364 (78.5%)
Rural	6595 (21.4%)	62 (26.5%)	1747 (21.5%)
Long‐term care	559 (1.8%)	22 (9.4%)	271 (3.3%)
Material deprivation, *n* (%)			
1 (most well‐off)	4251 (15.1%)	34 (15.6%)	1095 (14.8%)
2	4761 (16.9%)	41 (18.8%)	1193 (16.1%)
3	5612 (19.9%)	51 (23.4%)	1449 (19.5%)
4	6501 (23.1%)	51 (23.4%)	1709 (23.1%)
5 (most deprived)	7077 (25.1%)	41 (18.8%)	1967 (26.5%)
Missing	2591	16	698
Social deprivation, *n* (%)			
1 (most well‐off)	3849 (13.6%)	33 (15.1%)	1012 (13.7%)
2	4006 (14.2%)	31 (14.2%)	1016 (13.7%)
3	5351 (19.0%)	48 (22%)	1361 (18.4%)
4	6815 (24.2%)	51 (23.4%)	1811 (24.4%)
5 (most deprived)	8181 (29.0%)	55 (25.2%)	2213 (29.9%)
Missing	2591	16	698

Abbreviations: NMV‐*r*, nirmatrelvir/ritonavir; SD, standard deviation.

The mean Charlson Comorbidity Index score of the cohort was 1.8 (SD 2.5) (Table 3); those who were hospitalized were more likely to have a higher score than those who visited the ED only (hospitalized: 2.9 [SD 2.8]; ED only: 1.0 [SD 1.8]) (Table S5). The most common (> 10%) health conditions of interest that the cohort was living with were an immunocompromised status (35.9%), chronic kidney disease (16.6%), obesity (16.4%), diabetes and taking medication for the condition (16.1%), COPD (15.9%), and congestive heart failure (11.3%) (Table 3).

**TABLE 3:** Baseline clinical characteristics of the total cohort.

	Total	Medically eligible for NMV‐r	
		*n* = 8345	
		Received treatment	Did not receive treatment
	*n* = 30 793	*n* = 234	*n* = 8111
Charlson comorbidity index			
Overall score, mean (SD)	1.8 (2.5)	2.1 (2.2)	2.3 (2.6)
Category, *n* (%)			
0; no comorbidity	12 703 (41.3%)	51 (21.8%)	2350 (29%)
1–2; mild comorbidity	9791 (31.8%)	117 (50%)	3091 (38.1%)
3–4; moderate comorbidity	4238 (13.8%)	35 (15%)	1368 (16.9%)
≥ 5; severe comorbidity	4061 (13.2%)	31 (13.2%)	1302 (16.1%)
Health conditions, *n* (%)			
Type of condition			
Immunocompromised	11 041 (35.9%)	191 (81.6%)	5716 (70.5%)
Chronic kidney disease	5125 (16.6%)	26 (11.1%)	1594 (19.7%)
Obesity	5037 (16.4%)	45 (19.2%)	1514 (18.7%)
Diabetes, taking medication	4955 (16.1%)	51 (21.8%)	1792 (22.1%)
COPD	4901 (15.9%)	19 (8.1%)	865 (10.7%)
Congestive heart failure	3468 (11.3%)	38 (16.2%)	1504 (18.5%)
Pregnant	1220 (4.0%)	< 10 (~2.1%)	389 (4.8%)
Asthma	1159 (3.8%)	11 (4.7%)	444 (5.5%)
Number of above conditions			
0	17 886 (58.1%)	109 (46.6%)	3293 (40.6%)
1	7839 (25.5%)	76 (32.5%)	2756 (34%)
2	3547 (11.5%)	36 (15.4%)	1420 (17.5%)
≥ 3	1521 (4.9%)	13 (5.6%)	642 (7.9%)
Number of COVID‐19 vaccine doses received, *n* (%)			
0	6155 (20.0%)	46 (19.7%)	2560 (31.6%)
1	856 (2.8%)	< 10 (~2.1%)	257 (3.2%)
2	9969 (32.4%)	24–32 (~12.0%)	1981 (24.4%)
≥ 3	13 813 (44.9%)	155 (66.2%)	3313 (40.8%)
Prior COVID‐19 infection, *n* (%)	2215 (7.2%)	14 (6.0%)	586 (7.2%)

Abbreviations: COPD, chronic obstructive pulmonary disease; COVID‐19, coronavirus disease 2019; NMV‐*r*, nirmatrelvir/ritonavir; SD, standard deviation.

During the acute care encounter with COVID‐19, among those who were medically eligible for NMV‐r, the proportion who had an all‐cause death was 4.7% (received NMV‐r treatment) and 6.6% (did not receive NMV‐r treatment) (Table 4). Of those medically eligible for NMV‐r who were hospitalized, the mean length of hospital stay was 9.7 days (SD 11.1) in NMV‐r treated and 15.3 days (SD 25.9) in untreated, and the proportion admitted to the intensive care unit was 2.1% in NMV‐r treated and 7.6% in untreated (Table 4). Table S5 displays the acute care characteristics according to the acute care encounter type, whether NMV‐r was received or not, and the COVID‐19 diagnostic code location of the encounter.

**TABLE 4:** Acute care encounter characteristics of the total cohort.

	Total	Medically eligible for NMV‐r	
		*n* = 8345	
		Received treatment	Did not receive treatment
	*n* = 30 793	*n* = 234	*n* = 8111
All‐cause death, *n* (%)	1442 (4.7%)	11 (4.7%)	531 (6.6%)
Hospitalized, *n* (%)	13 112 (42.6%)	74 (31.6%)	4495 (55.4%)
LOS, days; mean (SD)	15.2 (26.2)	9.7 (11.1)	15.3 (25.9)
Admitted to ICU, *n* (%)	954 (7.3%)	< 10 (~2.1%)	340 (7.6%)
LOS, days; mean (SD)	15.9 (97.8)	11.1 (52.3)	17.4 (111.3)
Mechanically ventilated, *n* (%)	681 (5.2%)	< 10 (~2.1%)	231 (5.1%)

Abbreviations: ICU, intensive care unit; LOS, length of stay; NMV‐*r*, nirmatrelvir/ritonavir; SD, standard deviation.

## 4 Discussion

In this real‐world retrospective, observational, population‐based cohort study, the characteristics of adults who had an acute care COVID‐19 encounter during a timeframe that NMV‐r was available in Alberta, Canada (between January 2022 and March 2023) were described to assess medical eligibility and receipt of NMV‐r. Among adults who had an acute care encounter with COVID‐19 and were identified as being medically eligible for NMV‐r, most had not received this treatment within 30 days beforehand (97.2%); this potential care gap was observed regardless of medical eligibility criteria (immunocompromised status; age, health condition, and/or vaccination status; living in long‐term care or supportive living) that were in place at the time of presentation to acute care. These results highlight the need to identify and address barriers to early COVID‐19 oral antiviral treatment use for eligible high‐risk individuals to improve health outcomes and attendant health care resource use.

There is evidence on the efficacy and effectiveness of NMV‐r for the treatment of mild‐to‐moderately severe COVID‐19 from randomized controlled trials and real‐world observational studies. A recent rapid review with meta‐analyses and trial sequence analysis found that NMV‐r significantly decreased COVID‐19 hospitalization compared with placebo/no treatment (RR = 0.17 [95% CI: 0.10; 0.31]; *I* ^2^ = 77.2%; 2 randomized controlled trials, *n* = 3542); real‐world studies also showed NMV‐r significantly decreased COVID‐19 hospitalization (RR = 0.48 [95% CI: 0.37; 0.60]; *I* ^2^ = 95.0%; 11 real‐world studies, *n* = 1 421 398), and all‐cause mortality (RR = 0.24 [95% CI: 0.14; 0.34]; *I* ^2^ = 65%; 7 real‐world studies, *n* = 286 131) compared with placebo/no treatment [6]. An acknowledged caveat of these results is that the effectiveness of vaccine and infection induced immunity against current circulating strains may vary so the risk of progression to severe disease is not constant, thus the expected degree of benefit of therapy also may vary. Regardless, during the time frame of those studies, the evidence supported that NMV‐r decreases COVID‐19 hospitalizations and mortality among individuals with mild‐to‐moderately severe COVID‐19. A recent evidence synthesis concluded that generalizability of effectiveness to the Canadian population is needed [5]; additional contemporary evidence generation could address knowledge gaps.

In this study, most adults with an acute care COVID‐19 encounter who met medical eligibility criteria for NMV‐r during the study inclusion period did not receive treatment within 30 days prior to presentation, indicating a missed opportunity in care. Previous reports have shown that NMV‐r is underused in the target population of high risk individuals and suggest that low uptake may be partly driven by lack of awareness, an absence of health care provider recommendation of antiviral treatments for COVID‐19, misperceptions about treatments (effectiveness, adverse effects, requisite timing), and personal risk status for severe COVID‐19 illness, and challenges in timely diagnosis and drug access [9, 50, 51, 52]. If gaps are closed, based on current evidence of effectiveness, a proportion of COVID‐19 hospitalizations could be averted with attendant improvements in health and healthcare system burden. This study found that 4495 adults hospitalized with a COVID‐19 diagnosis over a 14.5 month period were, by medical history, potentially eligible for NMV‐r treatment but did not receive it. If they were treated, and assuming a conservative estimate of a 52% (RR = 0.48 [6]) reduction in COVID‐19 hospitalizations with NMV‐r treatment in those defined as high risk, up to 2337 hospitalizations may have been averted, with attendant improvement in other important patient outcomes. Improved public health communication strategies, education around individual COVID‐19 risk, and timely access to diagnostic testing and treatment may improve uptake of treatments that reduce severity of illness in at risk populations.

This study has several important strengths including the large size and population‐based design. However, this study is also subject to limitations that should be taken into consideration when interpreting results. Retrospective claims‐based studies use administrative data as opposed to medical records, thus there is a potential for misclassification of study cohorts or measures; validated case definitions were used, where available, to address this limitation. Ethnicity is not included in administrative health data and therefore eligibility criteria for NMV‐r that included Indigenous Persons, who represent 6.8% of the Alberta population [53], could not be identified; this limitation potentially underestimated those who had an acute care encounter with COVID‐19 and were medically eligible for NMV‐r and overestimated those who were not medically eligible. It could not be determined whether NMV‐r was taken according to recommendations, given that the onset and severity of symptoms are not available in administrative data, and the Pharmaceutical Information Network database only provides information on prescription medication dispensations rather than confirmed medication use.

### 4.1 Conclusions

Results from this real‐world study show that most adults with an acute care COVID‐19 encounter who were assessed as medically eligible for NMV‐r at the time of presentation to acute care had not received this outpatient treatment within 30 days beforehand in Alberta, Canada, suggesting a potential care gap. In addition to monitoring absolute risk of adverse outcomes and benefit of NMV‐r, identifying and overcoming barriers to outpatient COVID‐19 oral antiviral treatment among eligible high‐risk individuals is needed. If these care gaps are closed, individual health outcomes and strain on the acute healthcare system may be alleviated.

## Funding

This research study was funded by a grant (232402922) from the Pfizer‐Alberta Collaboration in Health with financial contributions from Pfizer Canada and the Government of Alberta that was evaluated and awarded by Alberta Innovates to the University of Alberta, with SK as the principal investigator. The funders had no role in the study design, data acquisition, analysis, and interpretation, or drafting of the manuscript. The funders were provided the opportunity to comment on the protocol and manuscript, with authors retaining the right to accept or reject comments or suggestions.

## Ethics Statement

Ethics approval was received from the University of Alberta Research Ethics Board (Pro00132799) that granted an exemption from requiring written informed consent (a waiver of consent was applied).

## Conflicts of Interest

The author(s) declared the following potential conflicts of interest with respect to the research and authorship of this report: S.A.H., K.V., J.R., K.M., P.U.N., T.W. and S.K. are members of the Alberta Real World Evidence Consortium (ARWEC) and the Alberta Drug and Technology Evaluation Consortium (ADTEC); these entities (comprised of individuals from the University of Alberta, University of Calgary, and Institutes of Health Economics) conduct research including academic investigator‐initiated industry‐funded studies (ARWEC) and government‐funded studies (ADTEC). Pfizer is the manufacturer of nirmatrelvir/ritonavir, and contributed research funding to the grant held by the University of Alberta, with S.K. as the principal investigator. In the past three years: the University of Alberta, with S.K. as the principal investigator, has received research funding from Moderna, a manufacturer of COVID‐19 vaccines; J.L. has received research funding from the Canadian Institutes for Health Research, Society for Healthcare Epidemiology of America, MSI Foundation, University of Calgary Department of Medicine and O’Brien Institute for Public Health, support for meeting attendance from the Canadian Institutes for Health Research, Society for Healthcare Epidemiology of America, and Research Canada, and a Pandemic EVIDENCE Collaboration fellowship from Kellogg College, Oxford University. No other Page 6 of 83 3 conflict of interest was declared. All authors of this study had complete autonomy over the design and execution of the study, as well as the content of this manuscript.

## Supporting information


**Data S1:** Supporting Information.
