# Supplementary material for: Eligibility for Nirmatrelvir/Ritonavir Among Adults With an Acute Care COVID‐19 Encounter: A Retrospective Cohort Study From Alberta, Canada
Source: Pharmacoepidemiol Drug Saf. 2025 Dec 25;35(1):e70302. doi: 10.1002/pds.70302 (PMC12741334; doi:10.1002/pds.70302)
Supplement: Supplementary file 1 — Data S1: Supporting Information. [file PDS-35-e70302-s001.docx]

Supplementary Table 1. Health conditions and their associated codes and weights included in the Charlson Comorbidity Index.

| Health condition | ICD-9-CM codes | ICD-10-CA codes | Weight |
| --- | --- | --- | --- |
| Myocardial infarction | 410, 412 | I21, I22, I25.2 | 1 |
| Congestive heart failure | 398, 402, 425, 428 | I09.9, I11.0, I13.0, I13.2, I25.5, I42.0, I42.5, I42.6, I42.7, I42.8, I42.9, I43, I50, P29.0 | 1 |
| Peripheral vascular disease | 440, 441, 443, 447, 557 | I70, I71, I73.1, I73.8, I73.9, I77.1, I79.0, I79.2, K55.1, K55.8, K55.9, Z95.8, Z95.9 | 1 |
| Cerebrovascular disease | 430, 431, 432, 433, 434, 435, 436, 437, 438 | G45, G46, I60, I61, I62, I63, I64, I65, I66, I67, I68, I69, H34.0 | 1 |
| Dementia | 290, 294, 331 | F00, F01, F02, F03, G30, F05.1, G31.1 | 1 |
| Chronic pulmonary disease | 416, 490, 491, 492, 493, 494, 495, 496, 500, 501, 502, 503, 504, 505 | J40, J41, J42, J43, J44, J45, J46, J47, J60, J61, J62, J63, J64, J65, J66, J67, I27.8, I27.9, J68.4, J70.1, J70.3 | 1 |
| Connective tissue disease | 446, 710, 714, 725 | M05, M32, M33, M34, M06, M31.5, M35.1, M35.3, M36.0 | 1 |
| Peptic ulcer disease | 531, 532, 533, 534 | K25, K26, K27, K28 | 1 |
| Mild liver disease | 070, 570, 571, 573 | B18, K73, K74, K70.0, K70.1, K70.2, K70.3, K70.9, K71.7, K71.3, K71.4, K71.5, K76.0, K76.2, K76.3, K76.4, K76.8, K76.9, Z94.4 | 1 |
| Moderate/severe liver disease | 456, 572 | K70.4, K71.1, K72.1, K72.9, K76.5, K76.6, K76.7, I85.0, I85.9, I86.4, I98.2 | 3 |
| Diabetes (without complication) | 250 | E10.0, E10.l, E10.6, E10.8, E10.9, E11.0, E11.1, E11.6, E11.8, E11.9, E12.0, E12.1, E12.6, E12.8, E12.9, E13.0, E13.1, E13.6, E13.8, E13.9, E14.0, E14.1, E14.6, E14.8, E14.9 | 1 |
| Diabetes (with complication) | 250 | E10.2, E10.3, E10.4, E10.5, E10.7, E11.2, E11.3, E11.4, E11.5, E11.7, E12.2, E12.3, E12.4, E12.5, E12.7, E13.2, E13.3, E13.4, E13.5, E13.7, E14.2, E14.3, E14.4, E14.5, E14.7 | 2 |
| Hemiplegia and paraplegia | 334, 342, 343, 344 | G81, G82, G04.1, G11.4, G80.1, G80.2, G83.0, G83.1, G83.2, G83.3, G83.4, G83.9 | 2 |
| Moderate or severe renal disease | 403, 582, 583, 585, 586, 588, V56 | N18, N19, N05.2, N05.3, N05.4, N05.5, N05.6, N05.7, N25.0, I12.0, I13.1, N03.2, N03.3, N03.4, N03.5, N03.6, N03.7, Z49.0, Z49.1, Z49.2, Z94.0, Z99.2 | 2 |
| Cancer | 140-165, 170-172, 174-176, 179-195, 200- 208, 238 | C00-C26, C30-C34, C37-C41, C43, C45-C58, C60-C76, C81-C85, C88, C90- C97 | 2 |
| Metastatic Carcinoma | 196, 197, 198, 199 | C77, C78, C79, C80 | 6 |
| HIV/AIDS | 042, 043, 044 | B20, B21, B22, B24 | 6 |

To be considered as having one of the listed diseases, a participant must have ≥1 hospitalization (associated ICD-10-CA code listed in any diagnostic field) or ≥2 physician claims (associated ICD-9-CM codes listed in any diagnostic field) of the corresponding ICD within ≤2-years. Abbreviations: HIV/AIDS = human immunodeficiency virus / acquired immunodeficiency syndrome; ICD-9-CM: International Classification of Disease – Version 9 – Clinical Modification (Alberta specific); ICD-10-CA: International Classification of Disease – Version 10 – Canadian Enhancement.

Supplementary Table 2. Eligibility criteria for nirmatrelvir/ritonavir in Alberta during the study inclusion period.

| Period | Eligibility | Medical Criteria | Notes |
| --- | --- | --- | --- |
| 1: January 18, 2022 to April 5, 2022^a^ | - PCR confirmed COVID-19 - ≤5 days of mild to moderate symptom onset - Meet medical criteria | Have no contraindications and:   - Aged 18+ AND have ≥1 health condition AND are unvaccinated - Aged 65+ AND are unvaccinated - Immunocompromised | From March 30, 2022 on^b^:   - Nirmatrelvir/ritonavir became available at more pharmacies in Alberta |
| 2: April 6, 2022 to  May 10, 2022^c^ | - PCR or rapid test (from May 5, 2022) confirmed COVID-19 - ≤5 days of mild to moderate symptom onset - Meet medical criteria | Have no contraindications and:   - Aged 18+ AND have ≥1 health condition/pregnant AND ≤1 COVID-19 vaccine dose - Aged 55+ or Indigenous and 45+ AND ≤1 COVID-19 vaccine dose - Immunocompromised - Living in long-term care or designated supportive living (level 4 and 4D) | From May 5, 2022 on^d^:   - Rapid test was sufficient; - All prescribers in Alberta could prescribe nirmatrelvir/ritonavir (included physicians, nurse practitioners, and some pharmacists) |
| 3: May 11, 2022 to January 19, 2023^e^ | - PCR or rapid test confirmed COVID-19; - ≤5 days of mild to moderate symptom onset - Meet medical criteria | Have no contraindications and:   - Aged 18+ AND have ≥1 health condition/pregnant AND ≤1 COVID-19 vaccine dose - Aged 55+ or Indigenous and 45+ AND ≤1 COVID-19 vaccine dose - Aged 60+ or Indigenous and 50+ AND have ≥1 health condition AND 2 COVID-19 vaccine doses - Aged 70+ or Indigenous and 60+ AND have ≥2 health conditions AND 3 COVID-19 vaccine doses - Immunocompromised - Living in long-term care or designated supportive living (level 4 and 4D) | |
| 4: January 20, 2023 to March 31, 2023^f^ | - PCR or rapid test confirmed COVID-19; - ≤5 days of mild to moderate symptom onset - Meet medical criteria | Have no contraindications and:   - Aged 18+ AND have ≥3 health conditions/pregnant - Aged 50+ or Indigenous and 40+ AND have ≥2 health conditions/pregnant - Aged 60+ or Indigenous and 50+ with ≥1 health condition - Immunocompromised - Living in long-term care or designated supportive living (level 4 and 4D) |  |
| Contraindications included the following medications^g^: amiodarone, apalutamide, bosentan, carbamazepine, clozapine, colchicine in patients with renal and/or hepatic impairment, digoxin, dronedarone, eplerenone, ergot derivatives (ergotamine, ergotamine combinations excl. psycholeptics, ergotamine combinations with psycholeptics, dihydroergotamine, dihydroergotamine combinations), flecainide, flibanserin, glecaprevir/pibrentasvir, ivabradine, lurasidone, mexiletine, phenobarbital, phenytoin, pimozide, propafenone, quinidine, ranolazine, rifampin, rifapentine, vorapaxar , St. John’s wort, calcineurin inhibitors (cyclosporine, tacrolimus), mTOR kinase inhibitors (sirolimus, everolimus), PDE5 inhibitors for pulmonary hypertension (sildenafil, tadalafil, vardenafil), anticoagulation, antiplatelet (apixaban, clopidogrel, rivaroxaban, ticagrelor, warfarin). | | | |

Pre-existing health conditions included diabetes and taking medication for treatment, obesity, chronic kidney disease, congestive heart failure, chronic obstructive pulmonary disease, and moderate-to-severe asthma. Abbreviations: COVID-19 = coronavirus disease 2019; PCR = polymerase chain reaction. ^a^Alberta Health. New at-home COVID-19 treatment on its way to Alberta. January 25, 2022; Available from: https://www.alberta.ca/release.cfm?xID=81782C40D5547-AD19-27C0-6D5D8405D1FD31A5. ^b^Alberta Health. Paxlovid to be available at more Alberta pharmacies. March 30, 2022; Available from: https://www.alberta.ca/release.cfm?xID=822359E14E00B-D965-BC47-BFD4C52D42540237. ^c^Alberta Health. Alberta expands access to fourth vaccine doses. April 6, 2022; Available from: https://www.alberta.ca/release.cfm?xID=822960A564540-9E17-A696-8D1B30A93143EF97#jumplinks-2. ^d^Alberta Health. Expanding Paxlovid accessibility. May 4, 2022; Available from: https://www.alberta.ca/release.cfm?xID=82493A9DBDEEC-B852-AA3F-6EFADF340E2ADA0A. ^e^Alberta Health Services. *Eligibility criteria for Paxlovid and Remdesivir expanded*. May 11, 2022; Available from: https://www.specialistlink.ca/assets/Outpatient-COVID-Treament-criteria-update-MEMO_May11.pdf. ^f^Alberta Health Services. *COVID-19, FAQs for health care providers*. January 20, 2023; Available from: https://www.albertahealthservices.ca/assets/info/ppih/if-ppih-covid-19-paxlovid-faq-patient.pdf. ^g^Alberta Blue Cross. *A reference guide: Paxlovid dispensing*. August 22, 2022; Available from: https://www.ab.bluecross.ca/pdfs/reference-guide-for-pharmacists-dispensing-paxlovid.pdf.

Supplementary Table 3. Case definitions used for identification of health conditions.

| Health condition | Algorithm | ICD-9-CM codes | ICD-10-CA and other codes |
| --- | --- | --- | --- |
| Asthma | ≥1 hospitalization or ≥3 ambulatory care / claims in ≤2 years | 493 | ICD-10: J45 |
| Congestive heart failure | ≥1 hospitalization or ≥2 claims in ≤2 years | 398, 402, 425, 428 | ICD-10: I09.9, I11.0, I13.0, I13.2, I25.5, I42.0, I42.5, I42.6, I42.7, I42.8, I42.9, I43, I50, P29.0 |
| Chronic kidney disease | ≥1 hospitalization or ≥3 claims ≤1 year OR mean eGFR <60 mL/min*1.73 m^2^ or mean albuminuria >30 mg/g over 1-year | 583-586, 592, 593.9 | ICD-10: N00-N23; eGFR and albuminuria laboratory results from lab data |
| COPD | ≥35 years with ≥1 hospitalization or claim in any years | 491-492, 496 | ICD-10: J41–J44 |
| Diabetes | ≥1 hospitalization or ≥2 claims in ≤2 years AND | 250 | ICD-10: E10-E14 |
| Taking medication for the health condition | ≥1 dispensation for a diabetic medication ≤3-months before the index date |  | ATC drug codes: see bottom of table |
| Immunocompromised | Had any of the following conditions or treatments: | | |
| Solid organ transplant | ≥1 healthcare encounter in any years | V42.0-V42.2, V42.4, V42.6- V42.9 | ICD-10: Z48.2, Z94 (not including: Z94.5, Z94.80, Z94.83, Z94.88, Z94.9); procedure code: 1.PC.85, 1.OA.85, 1.HY.85, 1.HZ.85, 1.GR.85, 1.OJ.85, 1.GT.85, 1.OK.85, 1.NK.85, 1.NP.85 |
| Allogenic / autologous bone marrow transplant | ≥1 healthcare encounter in any years | V42.81 | ICD-10: Z94.80; procedure code: 1.WY.19, 1.LZ.19.HH-U7, 1.LZ.19.HH-U8 |
| Receiving dialysis | ≥1 healthcare encounter in ≤2-years before the index date | V45.1, V56 | ICD-10: Z49, Z99.2; health services code: 13.99 A, B, C, D, O, or OA |
| Treated for tuberculosis | ≥1 hospitalization, ≥1 claim | 010-018 | ICD-10: A15-A19 |
| Sickle cell anemia | ≥1 hospitalization or ≥2 claims in ≤1 year | 282.6 | ICD-10: D57.0-D57.2, D57.8 |
| Immune system disorders | ≥1 hospitalization, ED, or claim OR ≥1 dispensation for an immunocompromising drug ≤1 year before index date | 279 | D80-D84, D89; ATC drug codes: see bottom of table |
| Cancer AND received chemotherapy or immunocompromising drug since Dec 2020 | ≥1 dispensation for chemotherapy (Cancer database) on/after December 1, 2020 OR ≥1 hospitalization or ≥2 claims in ≤2 years and received an immunocompromising drug on/after December 1, 2020. | 140-165, 170-172, 174-176, 179-195, 196-199, 200-208, 238 | ICD-10: C00-C26, C30-C34, C37-C41, C43, C45-C58, C60-C76, C77-C80, C81-C85, C88, C90- C97, D01.0-D01.3, D02.2, D05-D06, D07.5; Billing code for chemotherapy: 13.55A; procedure codes for chemotherapy: 1.ZZ.35.CA-M0, 1.ZZ.35.CA-M5, 1.ZZ.35.CA-M9, 1.ZZ.35.HA-M0, 1.ZZ.35.HA-M5, 1.ZZ.35.HA-M9, 1.ZZ.35.YA-M0, 1.ZZ.35.YA-M5, 1.ZZ.35.YA-M9. ATC immunocompromising drug codes: see bottom of table |
| Human immunodeficiency virus | ≥1 hospitalization or ≥3 claims in ≤3 years | 042-044 | ICD-10: B20-B24 |
| Other immunodeficiency conditions | ≥1 hospitalization |  | ICD-10: D70-D72, D73.0-D73.2, Q89.0; procedure codes for spleen removal: 1.OB.87, 1.OB.89 |
| Rheumatoid arthritis | ≥1 hospitalization or ≥3 claims with ≥1 by a specialist (rheumatologist, orthopedic surgeon, internal medicine) in ≤2 years | 714 | ICD-10: M05, M06 |
| Inflammatory bowel disease | ≥2 hospitalizations, ≥2 ambulatory care, or ≥4 claims in ≤2-years | 555, 556 | ICD-10: K50, K51 |
| Psoriasis | ≥1 hospitalization or ≥2 claims | 696 | ICD-10: L40.0-L40.4, L40.8, L40.9 |
| Psoriatic arthritis | ≥1 hospitalization or ≥3 claims for seronegative SpA (≥1 by a rheumatologist, internal medicine) and ≥1 claim for psoriasis | 696 (psoriasis), 721 (SpA) | ICD-10: L40.5, M07.0-M07.3, M09.0 |
| Multiple sclerosis | ≥1 hospitalization, or ≥5 claims from ambulatory care / claims in ≤2-years | 340 | ICD-10: G35 |
| Systematic autoimmune rheumatoid disease | ≥1 hospitalization or ≥3 claims | 710 | ICD-10: M32-M34, M35.0, M35.8, M35.9, M36.0 |
| Obesity | During the 10-year period before the index date: ICD code for obesity in the index acute care encounter; if not, then ≥1 hospitalization, ambulatory visit, or claim in any years (ICD or BMI fee-modifier code); excluded if no code listed after most recent dispensation for a weight-loss medication OR had bariatric surgery. | 278.0, V77.8 | ICD-10: E66; BMI modifier code: BMI^^^; ATC A08AB01, A08AA62; DIN 02437899; billing code: 55.8A, 55.8B 55.9A, or 56.93, or procedure code 1.NF.78.^^ |
| Pregnant | Female sex with: an ICD code for pregnancy in the index acute acre encounter OR ≥1 hospitalization, ambulatory visit, or claim for pregnancy ≤6 months before the index date (excluding delivery / abortion / miscarriage ICD codes between that visit and index date) OR ≥1 ICD code for delivery ≤6 months after index date. | Abortion/miscarriage: 632-637, 656.4; Delivery: 650-659, 660-665, V27; Other codes: V22, V23, 640-648 | Abortion/miscarriage: O00, O02.1, O03-O07, O36.4; Delivery: O60-O75, Z37; Other codes: Z34, Z35, O10-O16, O20-O29, O30-O48; Abortion Fee Codes: 87.0X, 87.2X; Abortion/miscarriage procedure codes: 5.CA.88, 5.CA.89, 5.CA.90, 5.CA.93; Delivery procedure codes: 5.MD.5, 5.MD.60; Maternal identifiers of birth records in vital statistics. |
| Pulmonary hypertension | ≥1 hospitalization | 416 | ICD-10: I27 |
| ATC codes for diabetic drugs: A10AB01, A10AB02, A10AB03, A10AB04, A10AB05, A10AB06, A10AC01, A10AC03, A10AD01, A10AD04, A10AD05, A10AE01, A10AE04, A10AE05, A10AE06, A10AE54, A10AE56, A10BA02, A10BB01, A10BB02, A10BB03, A10BB09, A10BB12, A10BB31, A10BD, A10BD03, A10BD04, A10BD07, A10BD09, A10BD10, A10BD11, A10BD13, A10BD15, A10BD16, A10BD19, A10BD20, A10BD21, A10BF01, A10BG01, A10BG02, A10BG03, A10BH01, A10BH03, A10BH04, A10BH05, A10BJ01, A10BJ02, A10BJ03, A10BJ04, A10BJ05, A10BJ06, A10BK01, A10BK02, A10BK03, A10BK04, A10BX02, A10BX03, A10BX16.  ATC codes for immunocompromising drugs: H02AA02, H02AB01, H02AB02, H02AB04, H02AB06, H02AB07, H02AB08, H02AB09, H02AB10, H02AB57, H02BX01, L01AA01, L01AA02, L01AA03, L01AA05, L01AA06, L01AA09, L01AB01, L01AB02, L01AC01, L01AD01, L01AD02, L01AD04, L01AX03, L01AX04, L01BA01, L01BA03, L01BA04, L01BA05, L01BB02, L01BB03, L01BB04, L01BB05, L01BB06, L01BB07, L01BC01, L01BC02, L01BC05, L01BC06, L01BC07, L01BC08, L01BC52, L01BC59, L01CA01, L01CA02, L01CA03, L01CA04, L01CB01, L01CB02, L01CD01, L01CD02, L01CD04, L01CE01, L01CE02, L01CX01, L01DA01, L01DB01, L01DB02, L01DB03, L01DB06, L01DB07, L01DB09, L01DC01, L01DC03, L01EA01, L01EA02, L01EA03, L01EA04, L01EA05, L01EA06, L01EB01, L01EB02, L01EB03, L01EB04, L01EB07, L01EC01, L01EC02, L01EC03, L01ED01, L01ED02, L01ED03, L01ED04, L01ED05, L01EE01, L01EE02, L01EE03, L01EE04, L01EF01, L01EF02, L01EF03, L01EG01, L01EG02, L01EH01, L01EH02, L01EH03, L01EJ01, L01EJ02, L01EK01, L01EL01, L01EL02, L01EL03, L01EM01, L01EM03, L01EN01, L01EN02, L01EN03, L01EX01, L01EX02, L01EX03, L01EX04, L01EX05, L01EX07, L01EX08, L01EX09, L01EX10, L01EX12, L01EX13, L01EX14, L01EX17, L01EX19, L01EX21, L01EX22, L01EX23, L01FA, L01FA01, L01FA02, L01FA03, L01FB01, L01FC01, L01FC02, L01FD01, L01FD02, L01FD03, L01FD04, L01FE01, L01FE02, L01FE03, L01FF01, L01FF02, L01FF03, L01FF04, L01FF05, L01FF06, L01FF07, L01FG01, L01FG02, L01FX02, L01FX03, L01FX04, L01FX05, L01FX06, L01FX07, L01FX08, L01FX09, L01FX10, L01FX12, L01FX13, L01FX14, L01FX17, L01FX18, L01X, L01XA01, L01XA02, L01XA03, L01XB01, L01XD01, L01XD03, L01XD04, L01XE, L01XF01, L01XG01, L01XG02, L01XG03, L01XH01, L01XH02, L01XJ01, L01XJ03, L01XK01, L01XK02, L01XK04, L01XL03, L01XL04, L01XL05, L01XL06, L01XL07, L01XX01, L01XX02, L01XX03, L01XX05, L01XX08, L01XX11, L01XX23, L01XX24, L01XX27, L01XX35, L01XX41, L01XX44, L01XX52, L01XX59, L01XX66, L01XX69, L01XX73, L01XX74, L01XX75, L01XY01, L01XY02, L02BA01, L02BA03, L02BB01, L02BB02, L02BB03, L02BB04, L02BB05, L02BB06, L02BG01, L02BG02, L02BG03, L02BG04, L02BG06, L02BX02, L02BX03, L03AA02, L03AA12, L03AA13, L03AB, L03AC01, L03AX03, L03AX12, L03AX13, L03AX16, L04AA02, L04AA03, L04AA04, L04AA06, L04AA10, L04AA13, L04AA15, L04AA18, L04AA21, L04AA23, L04AA24, L04AA25, L04AA26, L04AA27, L04AA29, L04AA31, L04AA32, L04AA33, L04AA34, L04AA36, L04AA37, L04AA38, L04AA40, L04AA42, L04AA43, L04AA44, L04AA47, L04AA48, L04AA50, L04AA51, L04AA52, L04AA54, L04AA56, L04AA59, L04AB01, L04AB02, L04AB04, L04AB05, L04AB06, L04AC01, L04AC02, L04AC03, L04AC05, L04AC07, L04AC08, L04AC10, L04AC11, L04AC12, L04AC13, L04AC14, L04AC16, L04AC17, L04AC18, L04AC19, L04AC21, L04AC22, L04AD01, L04AD02, L04AX01, L04AX02, L04AX03, L04AX04, L04AX06, V10XX02, X10XA53. | | | |

Case definitions were applied over a 5-year period before the index date, unless otherwise stated. Abbreviations: APHP = Alberta Perinatal Health Program; BMI = body mass index; COPD = chronic obstructive pulmonary disorder; eGFR = estimated glomerular filtration rate; ICD-9-CM: International Classification of Disease – Version 9 – Clinical Modification (Alberta specific); ICD-10-CA: International Classification of Disease – Version 10 – Canadian Enhancement.

Supplementary Table 4. Medical eligibility of the cohort for nirmatrelvir/ritonavir presented according to the acute care COVID-19 encounter diagnostic code location.

|  | Hospitalized  n=13,112 | |  | Emergency department only  n=17,681 | |
| --- | --- | --- | --- | --- | --- |
|  | COVID-19 diagnostic code location | |  | COVID-19 diagnostic code location | |
|  | Primary | Secondary |  | Primary | Secondary |
|  | n=6,200 | n=6,912 |  | n=13,943 | n=3,738 |
| *Medically eligible for nirmatrelvir/ritonavir, n (%)* | 2,213 (35.7%) | 2,356 (34.1%) |  | 3,010 (21.6%) | 766 (20.5%) |
| Overall |  |  |  |  |  |
| Received nirmatrelvir/ritonavir | 46 (2.1%) | 28 (1.2%) |  | 142 (4.7%) | 18 (2.3%) |
| Did not receive nirmatrelvir/ritonavir | 2,167 (97.9%) | 2,328 (98.8%) |  | 2,868 (95.3%) | 748 (97.7%) |
| According to eligibility criteria |  |  |  |  |  |
| Immunocompromised | 1,456 (65.8%) | 1,462 (62.1%) |  | 2,385 (79.2%) | 604 (78.9%) |
| Received nirmatrelvir/ritonavir | 36 (2.5%) | 19 (1.3%) |  | 122 (5.1%) | 14 (2.3%) |
| Did not receive nirmatrelvir/ritonavir | 1,420 (97.5%) | 1,443 (98.7%) |  | 2,263 (94.9%) | 590 (97.7%) |
| Age/health condition/vaccination status | 1,163 (52.6%) | 1,294 (54.9%) |  | 968 (32.2%) | 235 (30.7%) |
| Received nirmatrelvir/ritonavir | 11 (0.9%) | 10 (0.8%) |  | 32-40 (~3.7%) | <10 (~2.1%) |
| Did not receive nirmatrelvir/ritonavir | 1,152 (99.1%) | 1,284 (99.2%) |  | 928-936 (~96.3%) | 226-234 (~97.9%) |
| Long-term care/supportive living | 115 (5.2%) | 109 (4.6%) |  | 47 (1.6%) | 22 (2.9%) |
| Received nirmatrelvir/ritonavir | 11 (9.6%) | <10 (~4.6%) |  | <10 (~10.7%) | <10 (~22.7%) |
| Did not receive nirmatrelvir/ritonavir | 104 (90.4%) | 100-108 (~95.4%) |  | 38-46 (~89.3%) | 13-21 (~77.3%) |

Abbreviations: COVID-19 = coronavirus disease of 2019.

Supplementary Table 5. Characteristics of the cohort presented according to the index acute care COVID-19 encounter type overall, by those who received and did not receive nirmatrelvir/ritonavir within 30 days beforehand, and by the acute care COVID-19 encounter diagnostic code location.

|  | Hospitalized | | | | | | |  | Emergency department only | | | | | |
| --- | --- | --- | --- | --- | --- | --- | --- | --- | --- | --- | --- | --- | --- | --- |
|  | Total | | Received NMV-r ≤30 days  before the acute care visit | |  | COVID-19 diagnostic  code location | |  | Total | Received NMV-r ≤30 days  before the acute care visit | |  | COVID-19 diagnostic  code location | |
|  |  | | Yes | No |  | Primary | Secondary |  |  | Yes | No |  | Primary | Secondary |
|  | n=13,112 | | n=151 | n=12,961 |  | n=6,200 | n=6,912 |  | n=17,681 | n=301 | n=17,380 |  | n=13,943 | n=3,738 |
| *Demographic characteristics* |  | |  |  |  |  |  |  |  |  |  |  |  |  |
| Age, years |  | |  |  |  |  |  |  |  |  |  |  |  |  |
| Mean (SD) | 70 (19) | | 79 (14) | 70 (19) |  | 75 (15) | 65 (21) |  | 52 (20) | 64 (18) | 52 (20) |  | 52 (19) | 51 (20) |
| Category, n (%) |  | |  |  |  |  |  |  |  |  |  |  |  |  |
| 18-49 | 2,199 (16.8%) | | <10 (~3.3%) | 2,190-2,198 (~16.9%) |  | 447 (7.2%) | 1,752 (25.3%) |  | 8,613 (48.7%) | 69 (22.9%) | 8,544 (49.2%) |  | 6,631 (47.6%) | 1,982 (52.0%) |
| 50-59 | 1,099 (8.4%) | | <10 (~3.3%) | 1,090-1,098 (~8.4%) |  | 452 (7.3%) | 647 (9.3%) |  | 2,631 (14.9%) | 37 (12.3%) | 2,594 (14.9%) |  | 2,173 (15.6%) | 458 (12.2%) |
| 60-69 | 2,092 (16.0%) | | 21 (13.9%) | 2,071 (16.0%) |  | 1,018 (16.4%) | 1,074 (15.5%) |  | 2,616 (14.8%) | 67 (22.3%) | 2,549 (14.7%) |  | 2,107 (15.1%) | 509 (13.6%) |
| ≥70 | 7,722 (58.9%) | | 118 (78.1%) | 7,604 (58.7%) |  | 4,283 (69.1%) | 3,439 (49.8%) |  | 3,821 (21.6%) | 128 (42.5%) | 3,693 (21.2%) |  | 3,032 (21.7%) | 789 (21.1%) |
| Sex, n (%) |  | |  |  |  |  |  |  |  |  |  |  |  |  |
| Female | 6,363 (48.5%) | | 88 (58.3%) | 6,275 (48.4%) |  | 2,861 (46.1%) | 3,502 (50.7%) |  | 10,694 (60.5%) | 168 (55.8%) | 10,526 (60.6%) |  | 8,367 (60.0%) | 2,327 (62.3%) |
| Male | 6,749 (51.5%) | | 63 (41.7%) | 6,686 (51.6%) |  | 3,339 (53.9%) | 3,410 (49.3%) |  | 6,987 (39.5%) | 133 (44.2%) | 6,854 (39.4%) |  | 5,576 (40.0%) | 1,411 (37.7%) |
| Residence, n (%) |  | |  |  |  |  |  |  |  |  |  |  |  |  |
| Urban | 10,891 (83.1%) | | 127 (84.1%) | 10,764 (83%) |  | 4,925 (79.4%) | 5,966 (86.3%) |  | 13,307 (75.3%) | 197 (65.4%) | 13,110 (75.4%) |  | 10,268 (73.6%) | 3,039 (81.3%) |
| Rural | 2,221 (16.9%) | | 24 (15.9%) | 2,197 (17%) |  | 1,275 (20.6%) | 946 (13.7%) |  | 4,374 (24.7%) | 104 (34.6%) | 4,270 (24.6%) |  | 3,675 (26.4%) | 699 (18.7%) |
| Long-term care | 425 (3.2%) | | 26 (17.2%) | 399 (3.1%) |  | 216 (3.5%) | 209 (3.0%) |  | 134 (0.8%) | <10 (~1.7%) | 125-133 (~0.7%) |  | 82 (0.6%) | 52 (1.4%) |
| Material deprivation, n (%) |  | |  |  |  |  |  |  |  |  |  |  |  |  |
| 1 (most well-off) | 1,880 (16.0%) | | 24 (18.5%) | 1,856 (15.9%) |  | 811 (14.6%) | 1,069 (17.2%) |  | 2,371 (14.4%) | 44 (15.4%) | 2,327 (14.4%) |  | 1,786 (13.8%) | 585 (16.9%) |
| 2 | 1,879 (16.0%) | | 29 (22.3%) | 1,850 (15.9%) |  | 907 (16.3%) | 972 (15.6%) |  | 2,882 (17.5%) | 47 (16.5%) | 2,835 (17.6%) |  | 2,258 (17.4%) | 624 (18.0%) |
| 3 | 2,233 (19.0%) | | 28 (21.5%) | 2,205 (18.9%) |  | 1,025 (18.5%) | 1,208 (19.4%) |  | 3,379 (20.6%) | 56 (19.6%) | 3,323 (20.6%) |  | 2,655 (20.5%) | 724 (20.9%) |
| 4 | 2,653 (22.5%) | | 34 (26.2%) | 2,619 (22.5%) |  | 1,324 (23.8%) | 1,329 (21.4%) |  | 3,848 (23.4%) | 70 (24.6%) | 3,778 (23.4%) |  | 3,113 (24.0%) | 735 (21.2%) |
| 5 (most deprived) | 3,124 (26.5%) | | 15 (11.5%) | 3,109 (26.7%) |  | 1,486 (26.8%) | 1,638 (26.4%) |  | 3,953 (24.1%) | 68 (23.9%) | 3,885 (24.1%) |  | 3,155 (24.3%) | 798 (23.0%) |
| Missing | 1,343 | | 21 | 1,322 |  | 647 | 696 |  | 1,248 | 16 | 1,232 |  | 976 | 272 |
| Social deprivation, n (%) |  | |  |  |  |  |  |  |  |  |  |  |  |  |
| 1 (most well-off) | 1,646 (14.0%) | | 20 (15.4%) | 1,626 (14.0%) |  | 761 (13.7%) | 885 (14.2%) |  | 2,203 (13.4%) | 42 (14.7%) | 2,161 (13.4%) |  | 1,697 (13.1%) | 506 (14.6%) |
| 2 | 1,624 (13.8%) | | 19 (14.6%) | 1,605 (13.8%) |  | 742 (13.4%) | 882 (14.2%) |  | 2,382 (14.5%) | 42 (14.7%) | 2,340 (14.5%) |  | 1,891 (14.6%) | 491 (14.2%) |
| 3 | 2,071 (17.6%) | | 27 (20.8%) | 2,044 (17.6%) |  | 984 (17.7%) | 1,087 (17.5%) |  | 3,280 (20.0%) | 57 (20.0%) | 3,223 (20.0%) |  | 2,563 (19.8%) | 717 (20.7%) |
| 4 | 2,818 (23.9%) | | 30 (23.1%) | 2,788 (24.0%) |  | 1,345 (24.2%) | 1,473 (23.7%) |  | 3,997 (24.3%) | 70 (24.6%) | 3,927 (24.3%) |  | 3,192 (24.6%) | 805 (23.2%) |
| 5 (most deprived) | 3,610 (30.7%) | | 34 (26.2%) | 3,576 (30.7%) |  | 1,721 (31.0%) | 1,889 (30.4%) |  | 4,571 (27.8%) | 74 (26%) | 4,497 (27.8%) |  | 3,624 (27.9%) | 947 (27.3%) |
| Missing | 1,343 | | 21 | 1,322 |  | 647 | 696 |  | 1,248 | 16 | 1,232 |  | 976 | 272 |
|  |  | |  |  |  |  |  |  |  |  |  |  |  |  |
| *Clinical characteristics* |  | |  |  |  |  |  |  |  |  |  |  |  |  |
| Charlson Comorbidity Index |  | |  |  |  |  |  |  |  |  |  |  |  |  |
| Overall score, mean (SD) | 2.9 (2.8) | | 2.9 (2.2) | 2.9 (2.8) |  | 3.0 (2.7) | 2.8 (2.9) |  | 1.0 (1.8) | 1.9 (2.2) | 1.0 (1.8) |  | 1.0 (1.7) | 1.2 (2.0) |
| Category, n (%) |  | |  |  |  |  |  |  |  |  |  |  |  |  |
| 0; no comorbidity | 2,660 (20.3%) | | 16 (10.6%) | 2,644 (20.4%) |  | 951 (15.3%) | 1,709 (24.7%) |  | 10,043 (56.8%) | 86 (28.6%) | 9,957 (57.3%) |  | 7,969 (57.2%) | 2,074 (55.5%) |
| 1-2; mild comorbidity | 4,428 (33.8%) | | 62 (41.1%) | 4,366 (33.7%) |  | 2,262 (36.5%) | 2,166 (31.3%) |  | 5,363 (30.3%) | 136 (45.2%) | 5,227 (30.1%) |  | 4,310 (30.9%) | 1,053 (28.2%) |
| 3-4; moderate comorbidity | 2,898 (22.1%) | | 36 (23.8%) | 2,862 (22.1%) |  | 1,496 (24.1%) | 1,402 (20.3%) |  | 1,340 (7.6%) | 46 (15.3%) | 1,294 (7.4%) |  | 1,027 (7.4%) | 313 (8.4%) |
| ≥5; severe comorbidity | 3,126 (23.8%) | | 37 (24.5%) | 3,089 (23.8%) |  | 1,491 (24.0%) | 1,635 (23.7%) |  | 935 (5.3%) | 33 (11.0%) | 902 (5.2%) |  | 637 (4.6%) | 298 (8.0%) |
|  |  | |  |  |  |  |  |  |  |  |  |  |  |  |
| Health conditions, n (%) |  | |  |  |  |  |  |  |  |  |  |  |  |  |
| Type of condition |  | |  |  |  |  |  |  |  |  |  |  |  |  |
| Immunocompromised | 5,387 (41.1%) | | 84 (55.6%) | 5,303 (40.9%) |  | 2,791 (45.0%) | 2,596 (37.6%) |  | 5,654 (32.0%) | 186 (61.8%) | 5,468 (31.5%) |  | 4,439 (31.8%) | 1,215 (32.5%) |
| Chronic kidney disease | 3,585 (27.3%) | | 36 (23.8%) | 3,549 (27.4%) |  | 1,792 (28.9%) | 1,793 (25.9%) |  | 1,540 (8.7%) | 29 (9.6%) | 1,511 (8.7%) |  | 1,134 (8.1%) | 406 (10.9%) |
| Obesity | 2,165 (16.5%) | | 19 (12.6%) | 2,146 (16.6%) |  | 1,581 (25.5%) | 1,459 (21.1%) |  | 2,872 (16.2%) | 47 (15.6%) | 2,825 (16.3%) |  | 1,523 (10.9%) | 392 (10.5%) |
| Diabetes, taking medication | 3,040 (23.2%) | | 38 (25.2%) | 3,002 (23.2%) |  | 1,931 (31.1%) | 1,356 (19.6%) |  | 1,915 (10.8%) | 50 (16.6%) | 1,865 (10.7%) |  | 1,266 (9.1%) | 348 (9.3%) |
| COPD | 3,287 (25.1%) | | 44 (29.1%) | 3,243 (25%) |  | 1,368 (22.1%) | 1,316 (19.0%) |  | 1,614 (9.1%) | 44 (14.6%) | 1,570 (9.0%) |  | 562 (4.0%) | 222 (5.9%) |
| Congestive heart failure | 2,684 (20.5%) | | 32 (21.2%) | 2,652 (20.5%) |  | 1,119 (18.0%) | 1,046 (15.1%) |  | 784 (4.4%) | 23 (7.6%) | 761 (4.4%) |  | 2,309 (16.6%) | 563 (15.1%) |
| Pregnant | 584 (4.5%) | | <10 (~3.3%) | 575-583 (~4.5%) |  | 272 (4.4%) | 215 (3.1%) |  | 636 (3.6%) | <10 (~1.7%) | 627-635 (~3.6%) |  | 540 (3.9%) | 132 (3.5%) |
| Asthma | 487 (3.7%) | | <10 (~3.3%) | 478-486 (~3.7%) |  | 613 (9.9%) | 593 (8.6%) |  | 672 (3.8%) | 16 (5.3%) | 656 (3.8%) |  | 1,398 (10.0%) | 331 (8.9%) |
| Number of above conditions |  | |  |  |  |  |  |  |  |  |  |  |  |  |
| 0 | 5,100 (38.9%) | | 55 (36.4%) | 5,045 (38.9%) |  | 2,009 (32.4%) | 3,091 (44.7%) |  | 12,786 (72.3%) | 179 (59.5%) | 12,607 (72.5%) |  | 10,135 (72.7%) | 2,651 (70.9%) |
| 1 | 4,320 (32.9%) | | 55 (36.4%) | 4,265 (32.9%) |  | 2,195 (35.4%) | 2,125 (30.7%) |  | 3,519 (19.9%) | 88 (29.2%) | 3,431 (19.7%) |  | 2,767 (19.8%) | 752 (20.1%) |
| 2 | 2,471 (18.8%) | | 26 (17.2%) | 2,445 (18.9%) |  | 1,328 (21.4%) | 1,143 (16.5%) |  | 1,076 (6.1%) | 25-33 (~9.6%) | 1,047 (6.0%) |  | 832 (6.0%) | 244 (6.5%) |
| ≥3 | 1,221 (9.3%) | | 15 (9.9%) | 1,206 (9.3%) |  | 668 (10.8%) | 553 (8.0%) |  | 300 (1.7%) | <10 (~1.7%) | 291-299 (~1.7%) |  | 209 (1.5%) | 91 (2.4%) |
| Number of COVID-19 vaccine doses received, n (%) | | | |  |  |  |  |  |  |  |  |  |  |  |
| 0 | 3,126 (23.8%) | | 11-19 (~9.9%) | 3,105-3,115 (~24.0%) |  | 1,377 (22.2%) | 1,749 (25.3%) |  | 3,029 (17.1%) | 44-52 (~15.9%) | 2,983 (17.2%) |  | 2,469 (17.7%) | 560 (15.0%) |
| 1 | 374 (2.9%) | | <10 (~3.3%) | 365-373 (~2.8%) |  | 161 (2.6%) | 213 (3.1%) |  | 482 (2.7%) | <10 (~1.7%) | 473-481 (~2.7%) |  | 375 (2.7%) | 107 (2.9%) |
| 2 | 3,097 (23.6%) | | 13 (8.6%) | 3,084 (23.8%) |  | 1,359 (21.9%) | 1,738 (25.1%) |  | 6,872 (38.9%) | 45 (15.0%) | 6,827 (39.3%) |  | 5,396 (38.7%) | 1,476 (39.5%) |
| ≥3 | 6,515 (49.7%) | | 118 (78.1%) | 6,397 (49.4%) |  | 3,303 (53.3%) | 3,212 (46.5%) |  | 7,298 (41.3%) | 203 (67.4%) | 7,095 (40.8%) |  | 5,703 (40.9%) | 1,595 (42.7%) |
| Prior COVID-19 infection, n (%) | | 989 (7.5%) | <10 (~3.3%) | 943 (7.3%) |  | 460 (7.4%) | 492 (7.1%) |  | 1226 (6.9%) | 25 (8.3%) | 1,238 (7.1%) |  | 1,000 (7.2%) | 263 (7.0%) |
|  |  | |  |  |  |  |  |  |  |  |  |  |  |  |
| *Acute care characteristics* |  | |  |  |  |  |  |  |  |  |  |  |  |  |
| All-cause death, n (%) | 1,430 (10.9%) | | 19 (12.6%) | 1,411 (10.9%) |  | 642 (10.4%) | 788 (11.4%) |  | 12 (0.1%) | 0 (0%) | 12 (0.1%) |  | <10 (~0.04%) | <10 (~0.1%) |
| Hospitalized, n (%) |  | |  |  |  |  |  |  | - | - | - |  | - | - |
| LOS, days; mean (SD) | 15.2 (26.2) | | 11.5 (14.9) | 15.2 (26.3) |  | 12.0 (18.0) | 18.0 (31,6) |  | - | - | - |  | - | - |
| Admitted to ICU, n (%) | 954 (7.3%) | | 11 (7.3%) | 943 (7.3%) |  | 386 (6.2%) | 568 (8.2%) |  | - | - | - |  | - | - |
| LOS, days; mean (SD) | 15.9 (97.8) | | 15.1 (80.5) | 15.9 (98.0) |  | 18.0 (114.6) | 14.0 (79.8) |  | - | - | - |  | - | - |

Abbreviations: COPD = chronic obstructive pulmonary disease; COVID-19 = coronavirus disease 2019; NMV-r = nirmatrelvir/ritonavir, SD = standard deviation.


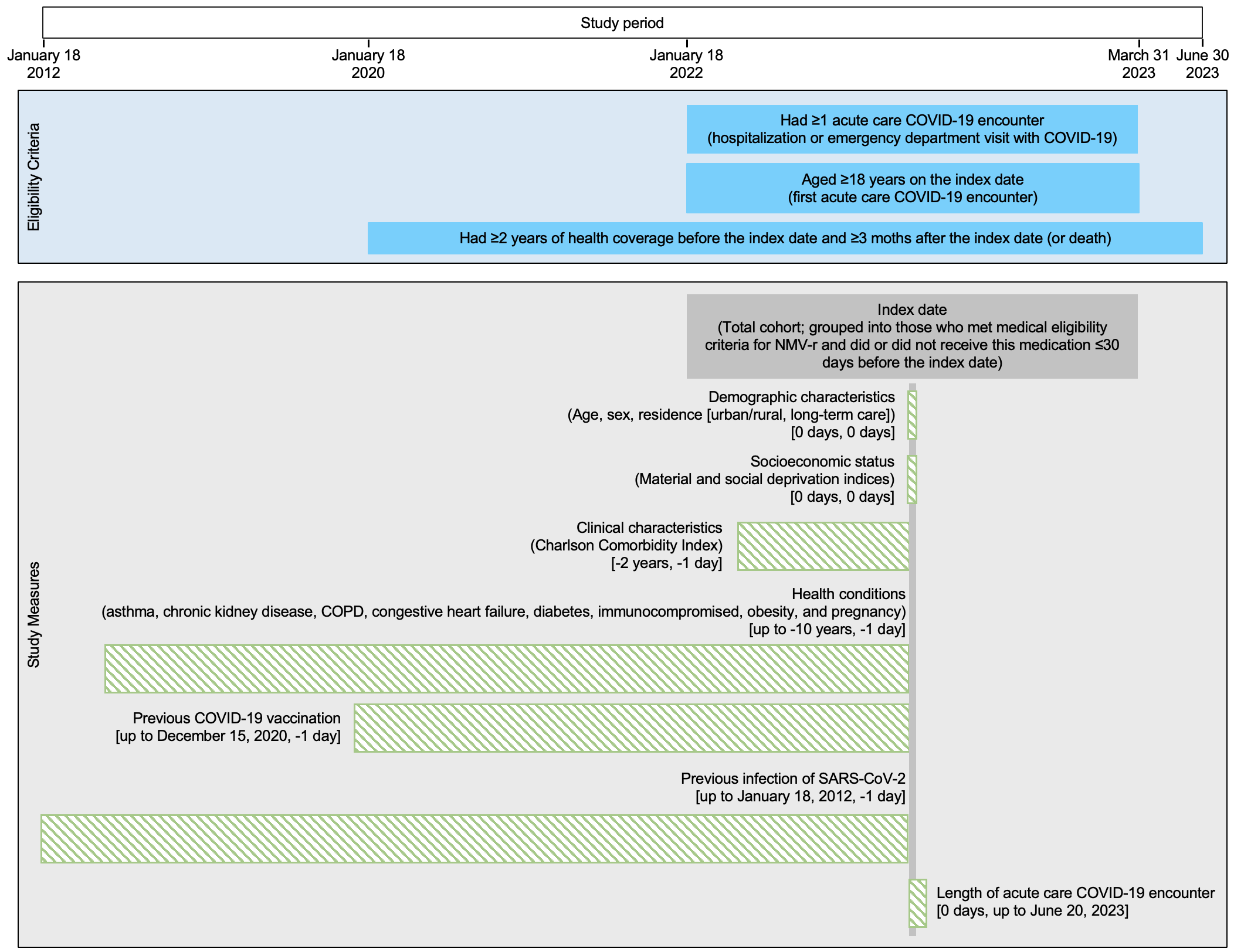


Supplementary Figure 1. Graphical depiction of the study design. Abbreviations: COVID-19 = coronavirus disease of 2019; SARS-CoV-2 = severe acute respiratory syndrome coronavirus 2.


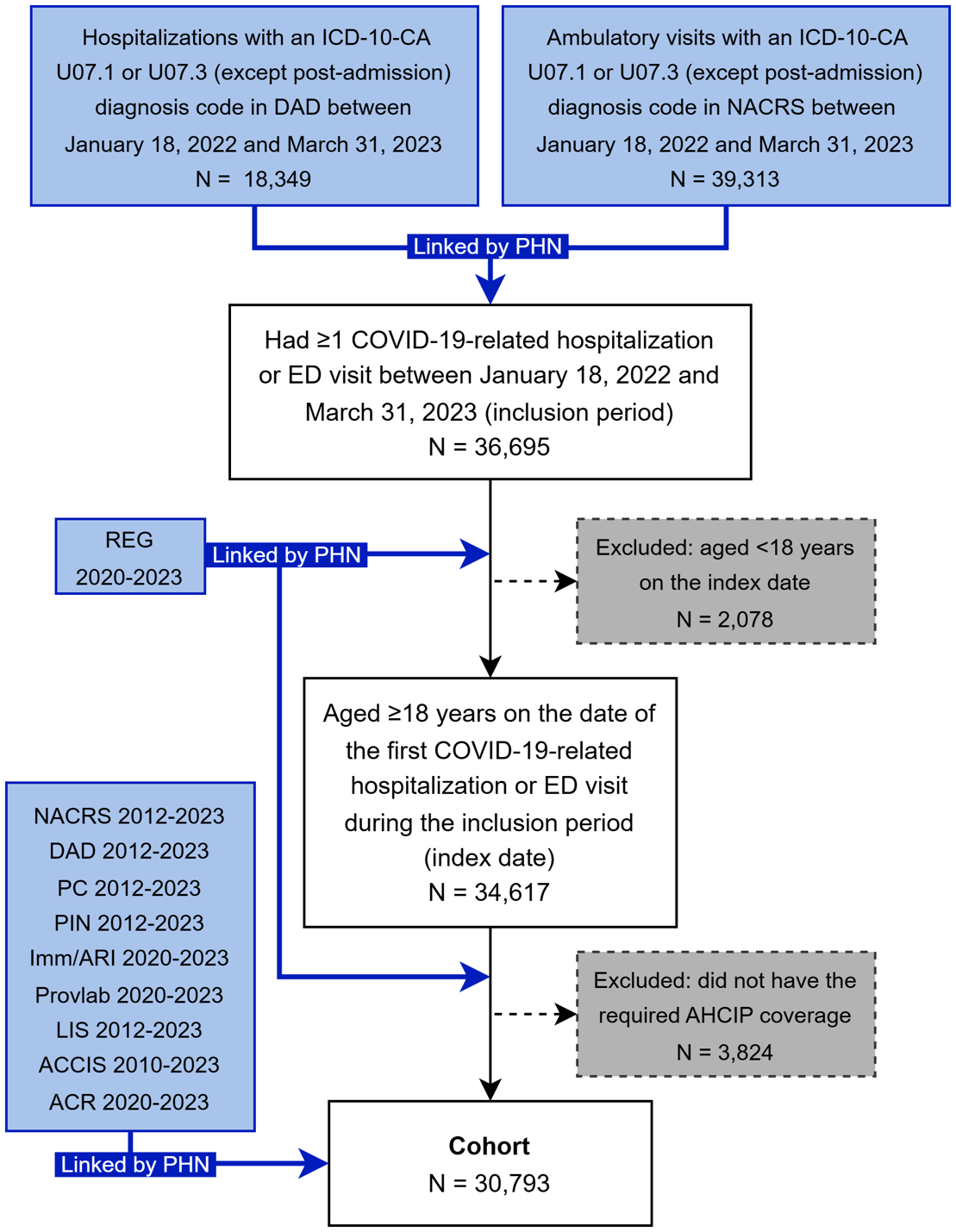


Supplementary Figure 2. Selection of the study cohort with data linkage shown. Abbreviations: ACCIS = Alberta Continuing Care Information System; ACR = Alberta Cancer Registry; AHCIP = Alberta Health Care Insurance Plan; COVID-19 = coronavirus disease of 2019; DAD = Discharge Abstract Database; ED = emergency department; ICD-10-CA: International Classification of Disease – Version 10 – Canadian Enhancement; Imm/ARI = Immunization and Adverse Reactions to Immunizations database; LIS = Laboratory Information System; NACRS = National Ambulatory Care Reporting System; PC = Practitioner Claims; PHN = Personal Health Number; PIN = Pharmaceutical Information Network; REG = Provincial Registry.
